# Genomic Variation across a Clinical Cryptococcus Population Linked to Disease Outcome

**DOI:** 10.1128/mbio.02626-22

**Published:** 2022-11-10

**Authors:** Poppy Sephton-Clark, Jennifer L. Tenor, Dena L. Toffaletti, Nancy Meyers, Charles Giamberardino, Síle F. Molloy, Julia R. Palmucci, Adrienne Chan, Tarsizio Chikaonda, Robert Heyderman, Mina Hosseinipour, Newton Kalata, Cecilia Kanyama, Christopher Kukacha, Duncan Lupiya, Henry C. Mwandumba, Thomas Harrison, Tihana Bicanic, John R. Perfect, Christina A. Cuomo

**Affiliations:** a Infectious Disease and Microbiome Program, Broad Institute of MIT and Harvard, Cambridge, Massachusetts, USA; b Division of Infectious Diseases, Department of Medicine, Duke University School of Medicine, Durham, North Carolina, USA; c Centre for Global Health, Institute of Infection and Immunity, St George's University of London, London, United Kingdom; d Clinical Academic Group in Infection, St George's University Hospital, London, United Kingdom; e Sunnybrook Health Sciences Centre, Toronto, Ontario, Canada; f Division of Infection and Immunity, University College London, London, United Kingdom; g UNC Project Malawi, University of North Carolina, Chapel Hill, North Carolina, USA; h Malawi-Liverpool-Wellcome Trust Clinical Research Programme, Blantyre, Malawi; i Tisungane Clinic, Zomba Central Hospital, Zomba, Malawi; University of Melbourne

**Keywords:** *Cryptococcus*, GWAS, aneuploidy, genome sequencing, sugar transporters, virulence

## Abstract

Cryptococcus neoformans is the causative agent of cryptococcosis, a disease with poor patient outcomes that accounts for approximately 180,000 deaths each year. Patient outcomes may be impacted by the underlying genetics of the infecting isolate; however, our current understanding of how genetic diversity contributes to clinical outcomes is limited. Here, we leverage clinical, *in vitro* growth and genomic data for 284 C. neoformans isolates to identify clinically relevant pathogen variants within a population of clinical isolates from patients with human immunodeficiency virus (HIV)-associated cryptococcosis in Malawi. Through a genome-wide association study (GWAS) approach, we identify variants associated with the fungal burden and the growth rate. We also find both small and large-scale variation, including aneuploidy, associated with alternate growth phenotypes, which may impact the course of infection. Genes impacted by these variants are involved in transcriptional regulation, signal transduction, glycosylation, sugar transport, and glycolysis. We show that growth within the central nervous system (CNS) is reliant upon glycolysis in an animal model and likely impacts patient mortality, as the CNS yeast burden likely modulates patient outcome. Additionally, we find that genes with roles in sugar transport are enriched in regions under selection in specific lineages of this clinical population. Further, we demonstrate that genomic variants in two genes identified by GWAS impact virulence in animal models. Our approach identifies links between the genetic variation in C. neoformans and clinically relevant phenotypes and animal model pathogenesis, thereby shedding light on specific survival mechanisms within the CNS and identifying the pathways involved in yeast persistence.

## INTRODUCTION

Cryptococcus neoformans is a pathogenic yeast that most commonly affects immunocompromised individuals, causing an estimated 180,000 deaths annually, with 75% of these occurring in sub-Saharan Africa. Cryptococcal infections, one of the leading causes of death in adults living with HIV/acquired immunodeficiency syndrome (AIDS), are especially problematic in low-income countries where, despite a widespread roll-out of antiretroviral therapy, the number of deaths due to opportunistic infections, such as cryptococcal meningitis, remains high (1). The infecting propagules of this pathogen generally enter human hosts via inhalation. From infections within the lung, C. neoformans may disseminate throughout the bloodstream and the central nervous system (CNS) of a susceptible patient, causing life-threatening meningitis (2). In a sample of health care systems across low-income countries, the 1-year mortality rate for individuals who develop cryptococcal meningitis is estimated to be 70% for those in care (uncertainty interval: 59 to 81%) (1). A better understanding of C. neoformans strain virulence and fitness within the host is necessary to improve patient outcomes and develop new treatment options.

While the majority of cryptococcosis cases are caused by Cryptococcus neoformans var. *grubii* (3), there are often high levels of genetic diversity within clinical populations of C. neoformans (4–7). Furthermore, isolates of the same multilocus sequence type (MLST) have been shown to cause infections that range in severity from mild to extreme (8). To examine how genetic variation contributes to virulence phenotypes, a recent study carried out a logistic regression analysis with 38 clinical C. neoformans isolates of the same sequence type to identify single nucleotide polymorphisms (SNPs) that are associated with patient survival, clinical parameters (including cytokine response, immune cell counts, and infection clearance), and *in vitro* data on absolute yeast growth and macrophage interactions (9). This study identified 40 candidate genes based on these association parameters, 6 of which (out of 17 genes tested) were important for survival in a murine model of C. neoformans infection. In a larger cohort of 230 C. neoformans samples from patients in South Africa, the isolate sequence type was associated with patient outcome, *in vitro* cerebrospinal fluid (CSF) survival, and phagocytosis response (10). Full scale genome-wide association studies (GWAS) have also examined how natural variation within a C. neoformans population differentiates clinical and environmental isolates, identifying loss-of-function variants present in clinical C. neoformans (VNB) populations that impact a transcription factor that is important for melanization, a well-studied virulence factor (11).

Furthermore, copy number variation, such as aneuploidy, has also been frequently identified within clinical populations of C. neoformans. Disomy of chromosome 1 is commonly reported for isolates exposed to azoles, and the higher copy number of two key genes, the *AFR1* transporter and the *ERG11* drug target, confer increased resistance to antifungals, such as fluconazole (12–14). Chromosome duplication as a result of *in vivo* passage has also been noted in clinical isolates (15–17), and the emergence of aneuploidy in this setting has been proposed as a mechanism by which both Cryptococcus and *Candida* species might rapidly adapt to high-stress environments (18, 19). In C. neoformans, aneuploidy is often transient, and passage under nonselective conditions allows for a reversion to euploidy (14, 17). In total, the aneuploidy of chromosomes 1, 2, 4, 6, 8, 9, 10, 12, 13, and 14 has been reported in C. neoformans (14, 16, 17, 20–23). Despite appearing consistently throughout clinical populations, the impacts of these other chromosomal aneuploidies are not yet well-understood.

To better understand how genetic variation among C. neoformans isolates contributes to infection outcomes in patients, we carried out genome-wide association studies (GWAS) with 266 C. neoformans clinical isolates from the VNI lineage, selected to reduce the confounding effects of population structure between lineages. In addition to comparing selected clinical data, all isolates were also measured for *in vitro* growth under diverse conditions. Through our GWAS approach, we identify two proteins that are associated with fungal burden in patients and demonstrate a connection to virulence in animal models. Additionally, we show that yeast survival within the rabbit CNS is dependent on a glycolytic gene identified by GWAS and also corroborate findings that patient outcome is highly correlated with fungal burden in the CNS. Partial and full chromosomal duplications are commonly detected within this clinical population, yet these aneuploidies reduce the fitness of C. neoformans under *in vitro* growth conditions.

## RESULTS

### The VNI lineage dominates clinical isolates and shows selection for sugar transporters.

**FIG 1:**
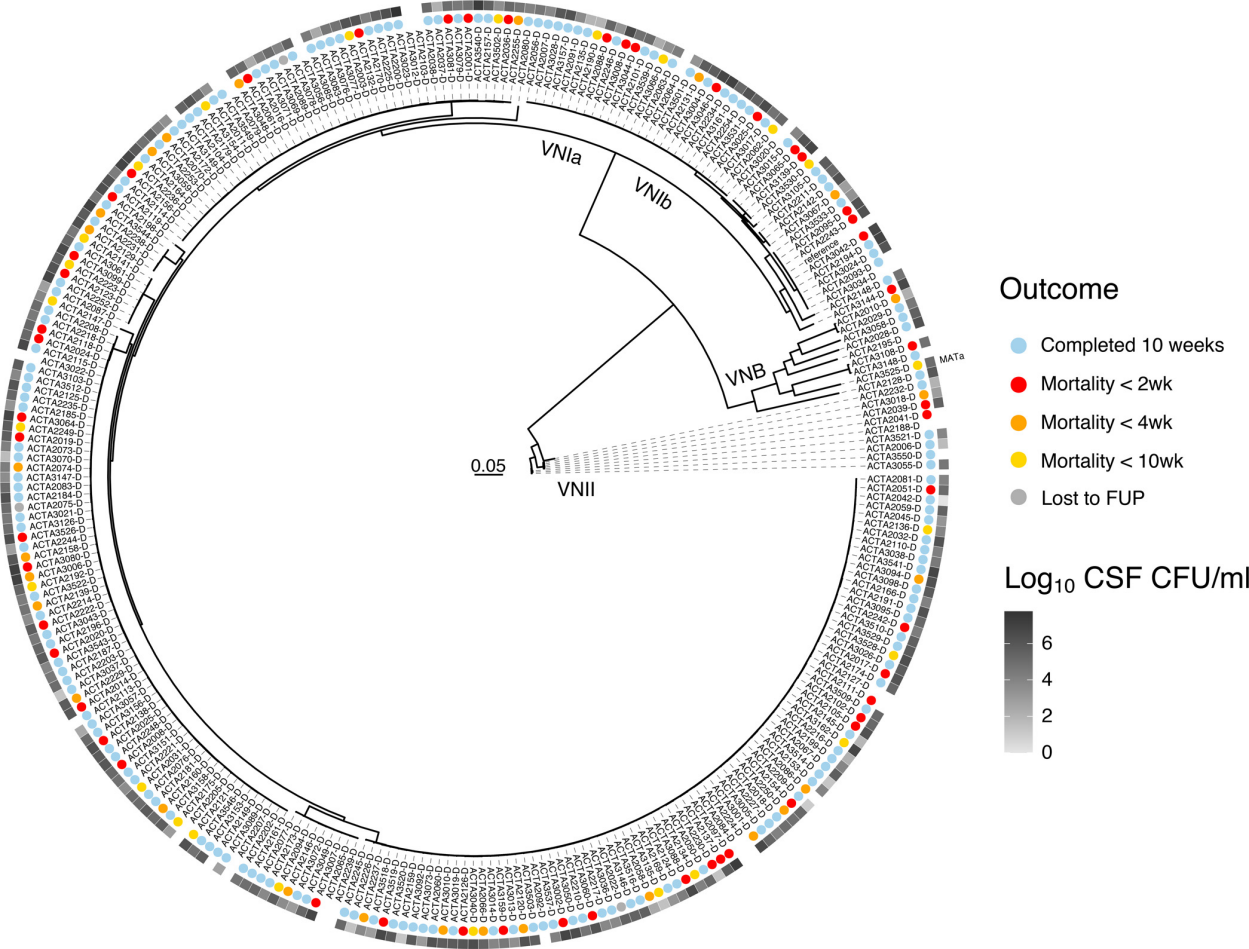
Maximum likelihood phylogeny of patient isolates, estimated from 72,258 segregating SNP sites, rooted to VNII. Isolates separate distinctly into VNI, VNB, and VNII, with all lineages having 100% bootstrap support. All isolates possess MAT α, except for ACTA3523 (highlighted). The colored circles correspond to patient survival. The grayscale squares indicate the patient fungal burden of cerebrospinal fluid prior to treatment, log_10_ CFU/mL.

To examine the variation within clinical populations, C. neoformans samples were isolated from HIV-infected patients as part of the ACTA trial and its human subject protocol (24), which evaluated the efficacy of fluconazole partnered with flucytosine, compared to amphotericin B combined with either fluconazole or flucytosine, as an induction therapy for cryptococcal meningitis. Baseline (preantifungal exposure) isolates were collected from three hospitals in Malawi between 2013 and 2016. We performed whole-genome sequencing on 344 isolates and removed isolates identified as Cryptococcus gattii (25), hybrid AD C. neoformans (4), diploid (2), or having low coverage (9) from the analysis. To examine the population structure, a maximum likelihood phylogeny was built based on segregating SNP sites (Fig. 1). Isolates can be clearly identified as VNI (266), VNII (9), and VNB (9), with the VNI isolates split into VNIa (217) and VNIb (26) with 100% bootstrap support; these recently described sublineages show strong evidence of separation (11). Of the two mating type loci found in Cryptococcus, mating type ɑ predominated among these isolates, with only one VNB isolate (ACTA3525) possessing the alternate *MAT*a. To assess recombination within the large VNI population, we calculated the linkage disequilibrium (LD) decay and found that the levels of decay for the VNI lineage (LD50 30 kb) (Fig. S1) were similar to those reported by Desjardins et al. (LD50 values for VNI, VNBI, and VNBII were less than 50 kb) (11). There is increased decay in the VNI population as a whole, compared to the individual VNIa and VNIb subgroups (LD50 of 110 kb for VNIb, LD50 not reached within 250 kb for VNIa), suggesting that VNIa and VNIb isolates do not recombine exclusively within their groups.

10.1128/mbio.02626-22.1 FIG S1 Linkage disequilibrium decay over 250 kb for lineages VNIa, VNIb, and VNI (VNIa + VNIb). VNI shows a 50% LD decay in 30 kb. Download FIG S1, PDF file, 0.1 MB. Copyright © 2022 Sephton-Clark et al. 2022 Sephton-Clark et al. https://creativecommons.org/licenses/by/4.0/ This content is distributed under the terms of the Creative Commons Attribution 4.0 International license.

To identify genomic regions under positive selection, we performed a composite likelihood ratio analysis (26). We found that regions with scores in the top 10% in more than one lineage (examining VNIa, VNIb, VNII, and VNB) include subtelomeric regions, centromeres, *ERG11*, and *AFR1* (Fig. S2). To examine whether genes within regions showing high selection scores are associated with shared functions, we performed a gene ontology (GO) enrichment analysis (hypergeometric test, false discovery rate [FDR] correction) on regions with selection scores in the top 5% (excluding centromeres). For the VNII isolates, we found that nucleotide excision repair was enriched in these regions (corrected *P* value of 6.77E−3). Sugar transport, including inositol transport, appeared to be robustly enriched in both the VNIa and VNB lineages (corrected *P* values of 1.07E−3 and 8.60E−3, respectively), supporting previous work that identified these functions as being under selection (Table S2) (11). To investigate the differences between functions under selection across lineages, we expanded our analysis of the VNII lineage to include genomic data from an additional 34 isolates for which we have whole-genome sequencing data (11, 22). In this expanded VNII cohort, we found that sugar transporters were enriched (corrected *P* value of 8.5E−4) in regions with selection scores in the top 5% (excluding centromeres). As genes with roles in hexose transport have been identified to be enriched in the subtelomeric regions of Cryptococcus sp. chromosomes (27), we examined the locations of sugar transporters that appeared under selection in VNI, VNB, and VNII. In total, 65% of sugar transporters in regions under selection fall into subtelomeric regions; however, only two of these genes are predicted to function specifically as hexose transporters (Table S2). Sugar transport and utilization have been identified as key to success in nutrient scarce environments, such as the CNS (23, 25, 28), important during interactions with amoebae, and important for roles in virulence and resistance to external stress (29–32).

10.1128/mbio.02626-22.2 FIG S2 Regions under selection per lineage, determined via CLR analysis. Highlighted regions are in the top 5% (orange) or top 10% (green) of all regions. Centromeric regions are highlighted in light grey. Region enrichment via hypergeometric testing for GO categories by lineage, with contributing genes and adjusted *P* values given. Download FIG S2, PDF file, 0.6 MB. Copyright © 2022 Sephton-Clark et al. 2022 Sephton-Clark et al. https://creativecommons.org/licenses/by/4.0/ This content is distributed under the terms of the Creative Commons Attribution 4.0 International license.

10.1128/mbio.02626-22.8 TABLE S2 Genes under selection for isolates of lineages VNI, VNB, and VNII. Download Table S2, XLSX file, 0.1 MB. Copyright © 2022 Sephton-Clark et al. 2022 Sephton-Clark et al. https://creativecommons.org/licenses/by/4.0/ This content is distributed under the terms of the Creative Commons Attribution 4.0 International license.

We also identified regions that were duplicated or deleted in these C. neoformans isolates via copy number variation analysis (Table S3). An 8 kb region was found to be duplicated in 43 of the VNIa isolates, containing 4 genes, including a sugar transporter (transporter classification database: 2.A.1.1, glycerol transport), a predicted noncoding RNA, a fungal specific transcription factor (Zn2Cys6, *SIP402*), and a short-chain dehydrogenase. A separate 34 kb region was found to be duplicated across 48 of the VNIa isolates, encoding 11 genes, including 3 dehydrogenase enzymes and 2 hydrolase enzymes. Duplicated regions unique to VNIb included an unannotated 19 kb region specific to 20 isolates and an 8 kb region that encodes 2 hypothetical proteins, duplicated across 21 VNIb isolates. Of the lineage-specific duplicated regions observed, all appeared to be specific to monophyletic groups within these lineages. Duplications of genes involved in resistance to azoles, such as *ERG11*, were found exclusively in VNII isolates; however, this finding did not correlate with an enhanced ability to grow in the presence of fluconazole at 64 μg/mL (Fig. S3). While these duplicated regions are not directly associated with our tested phenotypes, the duplication of regions containing genes such as *ERG11* and sugar transporters may still contribute to phenotypic variation that is relevant to clinical outcomes via the modulation of growth and virulence phenotypes when grown in alternate conditions.

10.1128/mbio.02626-22.3 FIG S3 Growth on fluconazole by lineage. Colony size (area, px) of isolates grown on fluconazole, 64 μg/mL, for 3 days. Download FIG S3, PDF file, 0.10 MB. Copyright © 2022 Sephton-Clark et al. 2022 Sephton-Clark et al. https://creativecommons.org/licenses/by/4.0/ This content is distributed under the terms of the Creative Commons Attribution 4.0 International license.

10.1128/mbio.02626-22.9 TABLE S3 Genes and gene annotations for duplicated regions identified via copy number variation analysis. Download Table S3, XLSX file, 0.1 MB. Copyright © 2022 Sephton-Clark et al. 2022 Sephton-Clark et al. https://creativecommons.org/licenses/by/4.0/ This content is distributed under the terms of the Creative Commons Attribution 4.0 International license.

### GWAS identifies multiple variants associated with fungal burden.

**FIG 2:**
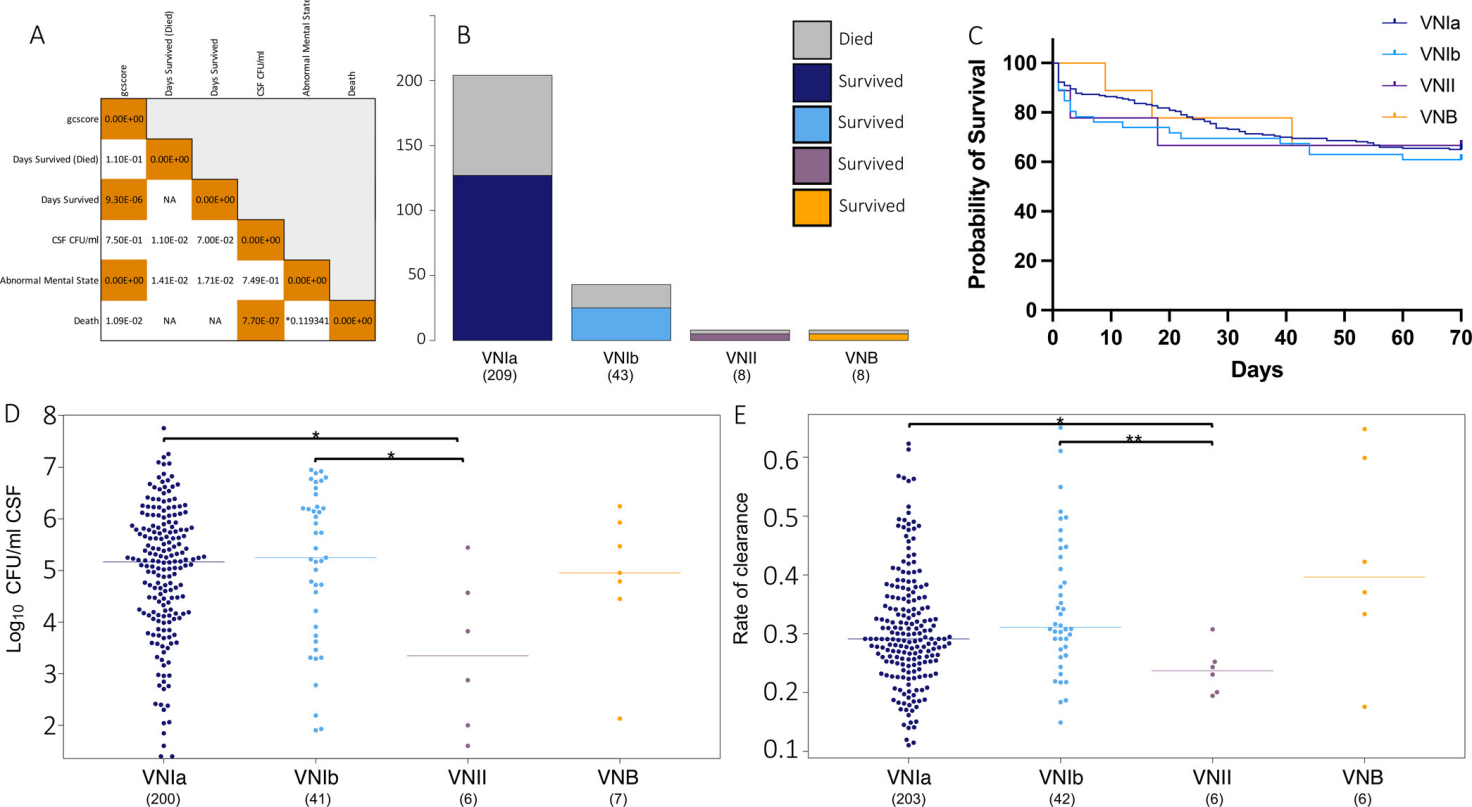
Clinical outcomes by lineage. (A) Correlation coefficient between clinical phenotypes, *P* < 0.0001 in orange. Asterisk indicates the phi coefficient. Days survived indicates the total number of days survived for all individuals. Days survived (Died) indicates the number of days survived only for individuals that died during the ACTA clinical trial. (B) Deaths (top, gray) and survival (bottom) of patients by the infecting isolate lineage. (C) Probability of survival, by lineage of infecting isolate. (D) CSF log_10_ CFU/mL (fungal burden) by infecting isolate lineage. Asterisk indicates *P* < 0.05 via a Wilcoxon test. (E) Rate of clearance (EFA) by the infecting isolate lineage. Displayed as −1(gradient). Asterisk indicates *P* < 0.05 and 0.01 via a Wilcoxon test.

Next, we used clinical data associated with these isolates to investigate the relationships between clinical factors, across lineages. We confirmed previous findings that mortality correlates strongly with a high baseline fungal burden (log_10_ CSF colony forming units [CFU]/mL, taken at the diagnosis of cryptococcal meningitis) (*P* value of 7.70E−7) (Fig. 2A), as shown in prior studies (33–35), and we also observed similar outcome ratios across lineages (log-rank test, *P* value of 0.916) (Fig. 2B and C), suggesting that there are no major lineage-specific differences in virulence. However, the low numbers of VNB and VNII infecting isolates used may limit our power to detect significant differences here. Additionally, we noted similar levels of baseline fungal burden and rates of clearance (EFA) between the VNIa and VNIb infecting isolates (Fig. 2D and E). The data suggest a reduction in the baseline fungal burden of 1.29E6 log_10_ CSF CFU/mL on average for the VNII isolates, compared to the VNI isolates (Wilcoxon test, *P* value of 0.024). However, due to the limited number of VNII isolates included, this finding should be confirmed with additional cases.

**FIG 3:**
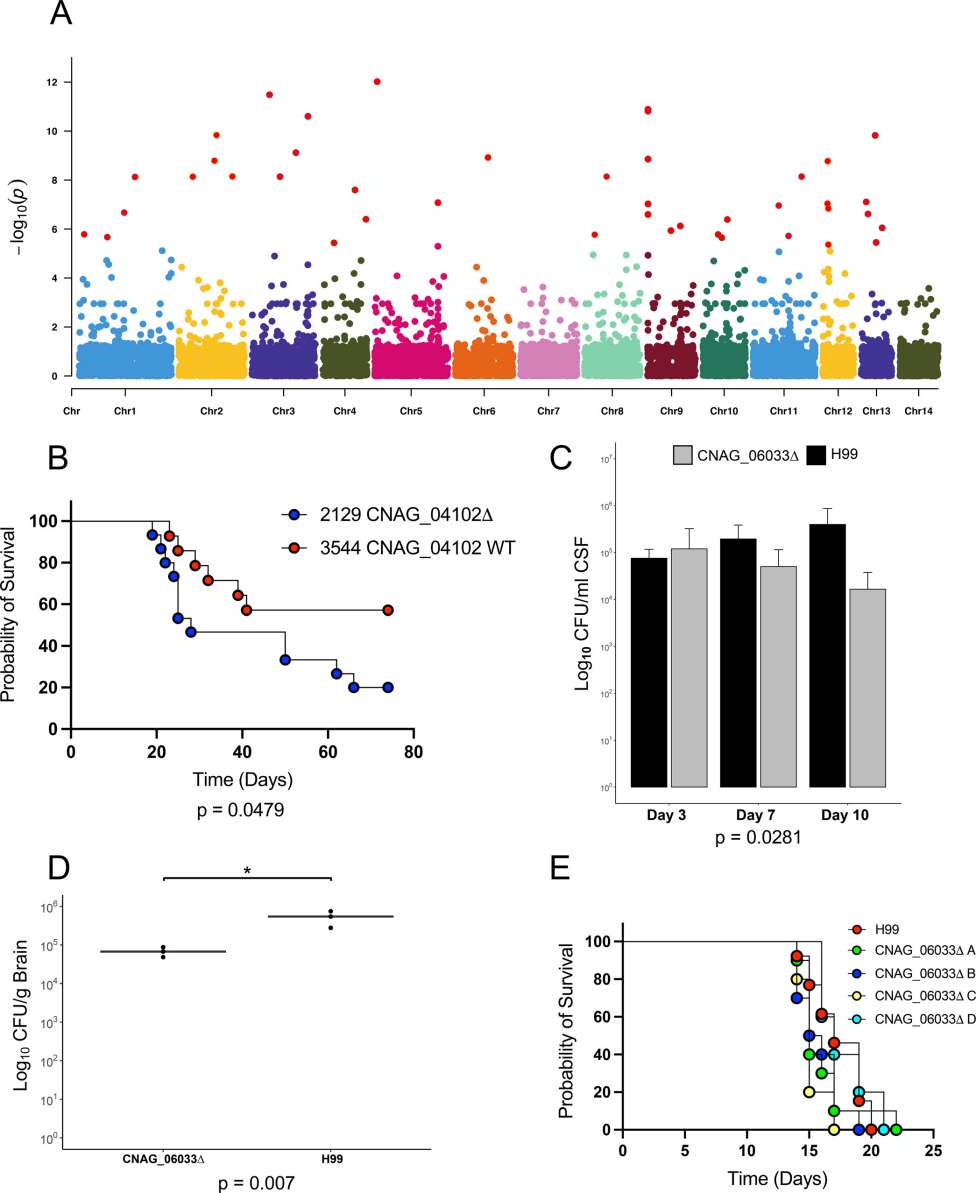
Impact on the virulence of genes containing variants significantly associated with fungal burden. (A) Manhattan plot displaying variants associated with a high fungal burden. Variants with an association score < 0.000005 (score test) are labeled in red. (B) Survival of mice infected with closely related ACTA isolates 2129 and 3544. 15 CD-1 mice were infected with approximately 5 × 10^4^ CFU by oropharyngeal aspiration. (C) Rabbit CSF CFU for the parental strain (H99) and for two independent CNAG_06033 mutant strains extracted on days 3, 7, and 10 postinfection. Three rabbits were infected per strain, with *n* = 3 per strain at days 3 and 7, and *n* = 2 per strain at day 10. (D) Rabbit log_10_ CFU/g for parental (H99) and one CNAG_06033 mutant strain, extracted from brain tissue (right lobe) at 10 days postinfection. (E) Survival of mice infected with the parental strain (H99) and four independent CNAG_06033 mutant strains. 5 CD-1 mice were infected with approximately 5 × 10^4^ CFU by oropharyngeal aspiration.

To determine whether the variation in baseline fungal burden, which appears to be well-distributed throughout this population (Fig. 1), is linked to a specific genetic component, we performed genome-wide association studies to identify variants associated with higher fungal loads, when taken as a continuous phenotype. We selected VNI isolates for this analysis not only because they represent the major genetic group present but also to avoid the confounding factors of population structure between lineages. This analysis revealed 53 variants that were significantly (GEMMA score test, *P* < 1.00E−6) associated with CSF fungal burden levels (Fig. 3A), 16 of which were predicted to result in a loss-of-function mutation. These variants impacted genes encoding 15 hypothetical proteins and 6 ncRNAs, and an additional 4 variants fell into noncoding centromeric regions (Table S4). Of the annotated genes impacted, 5 have been previously identified to modulate virulence phenotypes, and these include the SAGA histone acetylation complex *SGF29* (CNAG_06392), the protein S-acyl transferase *PFA4* (CNAG_03981), the calcineurin catalytic subunit *CNA1* (CNAG_04796), and the mitochondrial cochaperone *MRJ1* (CNAG_00938) that are required for virulence in the murine model, as well as the iron permease *FTR1* (CNAG_06242) that is required for capsule regulation (36–39). Additionally, 2 genes with variants are known to impact titan cell formation, and these include the multidrug transporter CNAG_04546 and the adenylate cyclase *CAC1* (CNAG_03202) (40, 41). A high proportion (28%) of variants with high GWAS scores (GEMMA score test, *P* < 1.00E–6) appeared in genes annotated as hypothetical proteins. We additionally performed GWAS to associate variants with mortality due to the correlation between mortality and high fungal burden. However, we did not observe any variants that were significantly associated with mortality, perhaps due to the multiple host factors that are known to impact this outcome, including the immune status of the host, raised intracranial pressure, duration of infection, and toxicities and adverse effects of antifungal drugs. For phenotype characterization, we decided to focus on genes impacted by variants that were associated with a higher fungal burden and were predicted to result in a loss-of-function or amino acid change within coding regions.

10.1128/mbio.02626-22.10 TABLE S4 Genes impacted by variants that are significantly associated with a high cerebrospinal fluid fungal burden. Download Table S4, XLSX file, 0.1 MB. Copyright © 2022 Sephton-Clark et al. 2022 Sephton-Clark et al. https://creativecommons.org/licenses/by/4.0/ This content is distributed under the terms of the Creative Commons Attribution 4.0 International license.

### Hypothetical proteins impact virulence in a strain- and background-dependent manner.

To determine whether the genes identified through our GWAS analysis of the fungal burden impact virulence in animal models, we tested a total of 10 gene deletion strains across murine and rabbit models. Previous work has shown that infection outcomes from human infections can be recapitulated in murine models (8). Furthermore, rabbit models have proven useful in evaluating CNS infections, as the fungal burden within the CNS can be determined via the longitudinal collection of CSF (42). The most striking result from our GWAS analysis was a set of 5 different variants in the same hypothetical protein, CNAG_04102, with the highest scoring frameshift variant within this gene being the third most significant overall (GEMMA score test, *P* = 1.30E−11). There were 12 isolates with natural loss-of-function variants in CNAG_04102. To determine whether these variants impact virulence, we chose two closely related clinical isolates (ACTA2129 and ACTA3544), one with and one without a frameshift in CNAG_04102, and tested them in a murine model. The isolate with a frameshift in CNAG_04102 showed significantly increased rates of mortality (*P* = 0.0479) (Fig. 3B), compared to the control isolate. However, when two independent deletions of this gene were generated in an H99 background and tested in a murine model, we observed no significant impact on virulence (Fig. S6), highlighting the complex impacts of these variants in a background-specific manner. CNAG_04102 contains a Kyakuja-Dileera-Zisupton (KDZ) superfamily motif (pfam18758), which has been found within species from Basidiomycota, Mucoromycotina, *Rhizophagus*, and *Allomyces* (43), with CNAG_04102 homologs containing this motif having been found in Cryptococcus gattii and in Cryptococcus floricola. The KDZ motif is also commonly located within TET/JBP genes, which are involved in genomic organization and epigenetic regulation (44), suggesting a role for gene expression regulation. A second hypothetical protein, CNAG_05608, displayed missense variants that were associated with fungal burden in 22 isolates. An available CNAG_05608 deletion strain in the CMO26 KN99 background (45) showed reduced virulence within a murine model, compared to wild-type CMO26 (log-rank test, *P* = 0.0154) (Fig. S6). However, when two independent deletions of CNAG_05608 were tested in an H99 background, we saw no difference in virulence, again highlighting the impact of strain background in virulence phenotypes (Fig. S6). While CNAG_05608 is annotated as a hypothetical protein, this gene is predicted to contain a single transmembrane domain and has homologs in Cryptococcus gattii, Cryptococcus amylolentus, *Kwoniella* species, and *Wallemia* species. Furthermore, this gene is upregulated during growth in both murine and monkey lungs (46), and it is slightly downregulated when grown in the presence of glucose (47), suggesting a role during infection.

10.1128/mbio.02626-22.6 FIG S6 Survival of mice infected with parental strain (H99/CMO26) or a deletion strain. Five CD-1 mice were infected with approximately 5 × 10^4^ CFU by oropharyngeal aspiration per strain. (A) Survival of mice infected with the parental strain (H99) and a CNAG_00544 mutant strain. (B) Survival of mice infected with the parental strain (CMO26) and a CNAG_02777 mutant strain. (C) Survival of mice infected with the parental strain (CMO26) and a CNAG_05119 mutant strain. (D) Survival of mice infected with the parental strain (H99) and two independent CNAG_04102 mutant strains. (E) Survival of mice infected with the parental strain (CMO26) and a CNAG_04548 mutant strain. (F) Survival of mice infected with the parental strain (CMO26) and a CNAG_03754 mutant strain. (G) Survival of mice infected with the parental strain (CMO26) and a CNAG_03161 mutant strain. (H) Survival of mice infected with the parental strain (H99/CMO26) and three independent CNAG_05608 mutant strains in the CMO26 background (C) and in the H99 background (A, B). Download FIG S6, PDF file, 0.3 MB. Copyright © 2022 Sephton-Clark et al. 2022 Sephton-Clark et al. https://creativecommons.org/licenses/by/4.0/ This content is distributed under the terms of the Creative Commons Attribution 4.0 International license.

### Sugar transport and metabolism impact persistence in a rabbit CNS infection model.

In testing for the association of loss-of-function variants with fungal burden, the most highly significant variant was a frameshift in the phosphofructokinase gene, CNAG_06033 (pfkB) (GEMMA score test, *P* = 1.69E−09). The deletion mutants resulting from four independent deletions of CNAG_06033 in an H99 background displayed a trend toward increased virulence in a murine model, compared to H99 (17 day median survival for H99, 15.5 day median survival for the CNAG_06033 deletions), though the result was not statistically significant (log-rank test, *P* = 0.0637) (Fig. 3). Two mice with CFU counts for H99 were excluded at day 42, as they were identified as outliers via a ROUT analysis. Paradoxically, two independent deletion mutants of this gene displayed a significantly decreased CSF burden within the rabbit model (3 New Zealand White male rabbits), compared to the parental strain, with the CSF levels (log_10_ CFU/mL counts) being dependent on both the infecting strain and the number of days postinfection. The CSF loads were comparable across three rabbits for H99 and for the two CNAG_06033 deletion strains at 3 days postinfection, but they decreased significantly for the CNAG_06033 deletion strains at days 7 and 10, in contrast to H99, which showed an increased CSF load over time (repeated measures analysis, *P* = 0.028) (Fig. 3C). This highlights the need for efficient glycolysis within the mammalian CSF, as the disruption of both early and late glycolysis regulatory genes, phosphofructokinase (CNAG_06033), and pyruvate kinase (25) reduces C. neoformans growth within the rabbit CSF. Congruently, the fungal burdens within the brains of rabbits infected with a CNAG_06033 deletion strain also appeared to be significantly reduced, compared to those infected with H99 (*t* test, *P* = 0.007) (Fig. 3D). Loss-of-function variants within CNAG_06033 have been shown to emerge over the course of *in vivo* passage in CSF during human infection and relapse (15), suggesting a role for the loss of CNAG_06033 in adaptation to a specific host. However, in an acute rabbit infection model, the loss of CNAG_06033 results in lower survival for C. neoformans. This result is consistent with prior work that tested deletions of other genes that were directly involved in glycolysis; the loss of pyruvate kinase (*pyk1Δ*) resulted in decreased persistence in the rabbit CSF, but dissemination was unperturbed in a murine model (25). To better understand the requirements of glycolysis and sugar transport in the efficient survival of C. neoformans within the CSF, we identified significant variants in additional genes involved in these pathways. A predicted xylose transporter, CNAG_05324, contained a frameshift variant that was present in 33 isolates (GEMMA score test, *P* = 4.00E−07). In preliminary experiments, the deletion of CNAG_05324 in a H99 background led to an increase in the CSF load in one rabbit, compared to its H99 control. However, additional experiments are required to confirm these results (Fig. S4A). Given the predicted role of this gene as a xylose transporter and the presence of xylose in the cryptococcal capsule, we undertook preliminary phenotypic capsule screening of the CNAG_05324 deletion strain. A capsule analysis of this deletion mutant via cultivation in capsule-inducing media and India ink staining revealed a significant increase in capsule thickness (Welch’s t test, *P* = 3.9E−14) (Fig. S4B), suggesting a role for CNAG_05324 in the modulation of capsule size. Previous work has shown that the modulation of xylose transport and xylosylation can drastically alter virulence, capsule size, and immune evasion (48, 49), highlighting this capsular mechanism as an area for further exploration.

10.1128/mbio.02626-22.4 FIG S4 Capsule and virulence regulation of CNAG_05324. (A) Rabbit CSF load, in log_10_ CFU/mL, on days 3, 7, and 10, per rabbit (individual lines) infected with either H99 (blue) or the CNAG_05324 deletion strain (pink). (B) Cell size and capsule diameter at 48 h of growth in a capsule inducing media for H99 and for the CNAG_05324 deletion strain. Download FIG S4, PDF file, 0.2 MB. Copyright © 2022 Sephton-Clark et al. 2022 Sephton-Clark et al. https://creativecommons.org/licenses/by/4.0/ This content is distributed under the terms of the Creative Commons Attribution 4.0 International license.

### Aneuploidy is common and slows growth.

**FIG 4:**
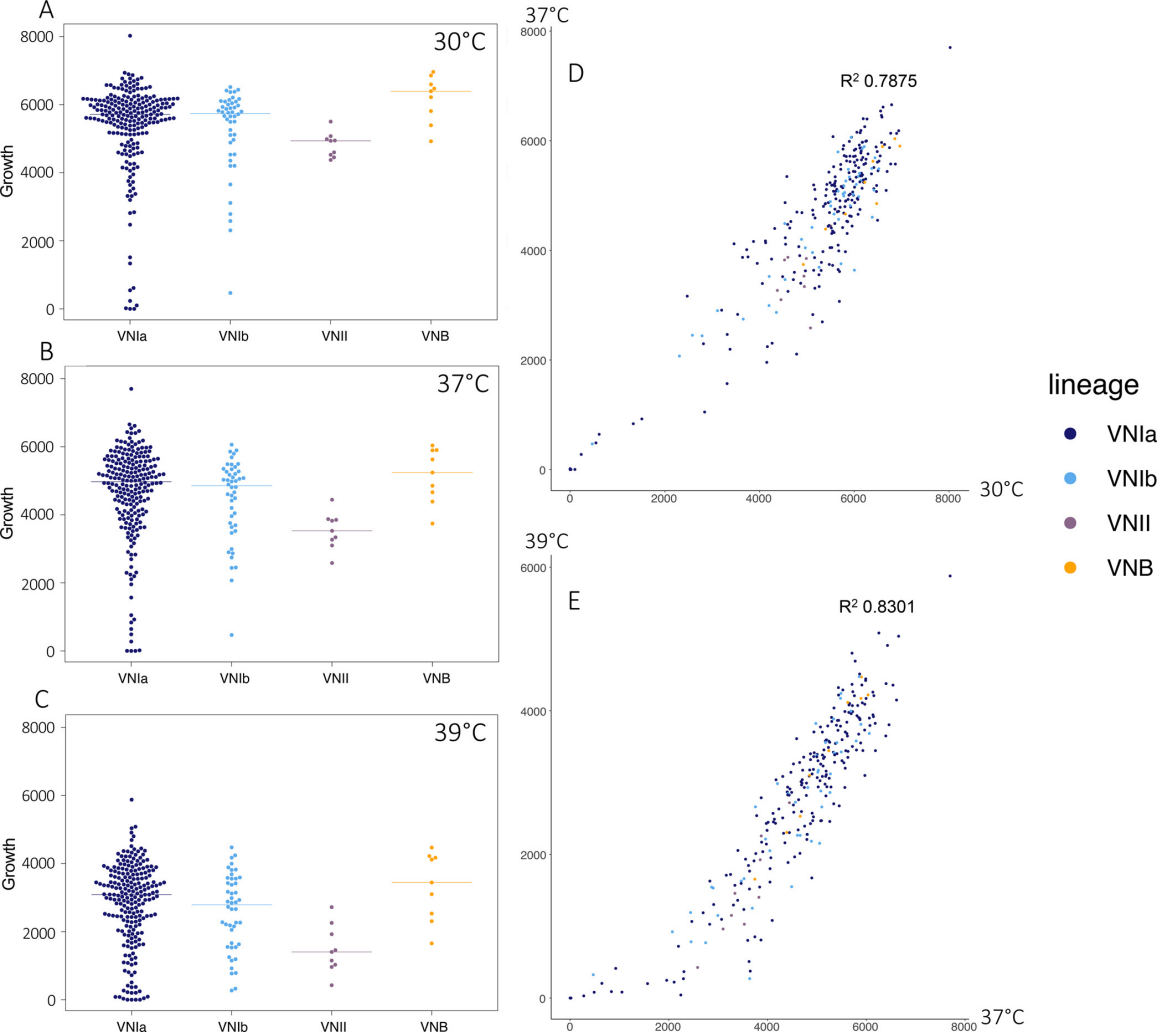
Isolate growth and phenotype correlation. Colony size (area, px) of isolates grown on YPD at (A) 30°C, (B) 37°C, and (C) 39°C. Correlation of isolate growth (area, px) on YPD, with the axes corresponding to the colony size when grown at (D) 30°C and 37°C or at (E) 37°C and 39°C. Colors correspond to the isolate lineage: VNIa (dark blue), VNIb (light blue), VNII (purple), VNB (orange).

To determine how natural variation might affect growth and other clinically relevant phenotypes, we performed *in vitro* phenotyping of isolates. Isolates displayed a range of growth levels on rich media (YPD) at 30°C, 37°C, and 39°C (Fig. 4A–C), with colony size showing strong correlation across conditions and replicates (Fig. 4D and E) (replicate per condition, R^2^ > 0.8). To determine whether this variation is linked to a specific genetic component, we performed GWAS to identify variants associated with increased and decreased colony size. Significantly associated with the rapid growth of large colonies on YPD were loss-of-function variants in CNAG_06637 (UBP8 ubiquitin-specific protease, a component of the SAGA complex), CNAG_03818 (sensory transduction histidine kinase), and CNAG_10082 (tRNA threonine) (GEMMA score test, *P* < 1.00E−6). A single loss-of-function variant was found to be significantly associated with decreased colony size, with a frameshift in the dolichyldiphosphatase encoding gene, CNAG_03014 (GEMMA score test, *P* = 9.90E−12). Naturally occurring loss-of-function variants such as these may play a role in the fitness variation observed between clinical isolates.

**FIG 5:**
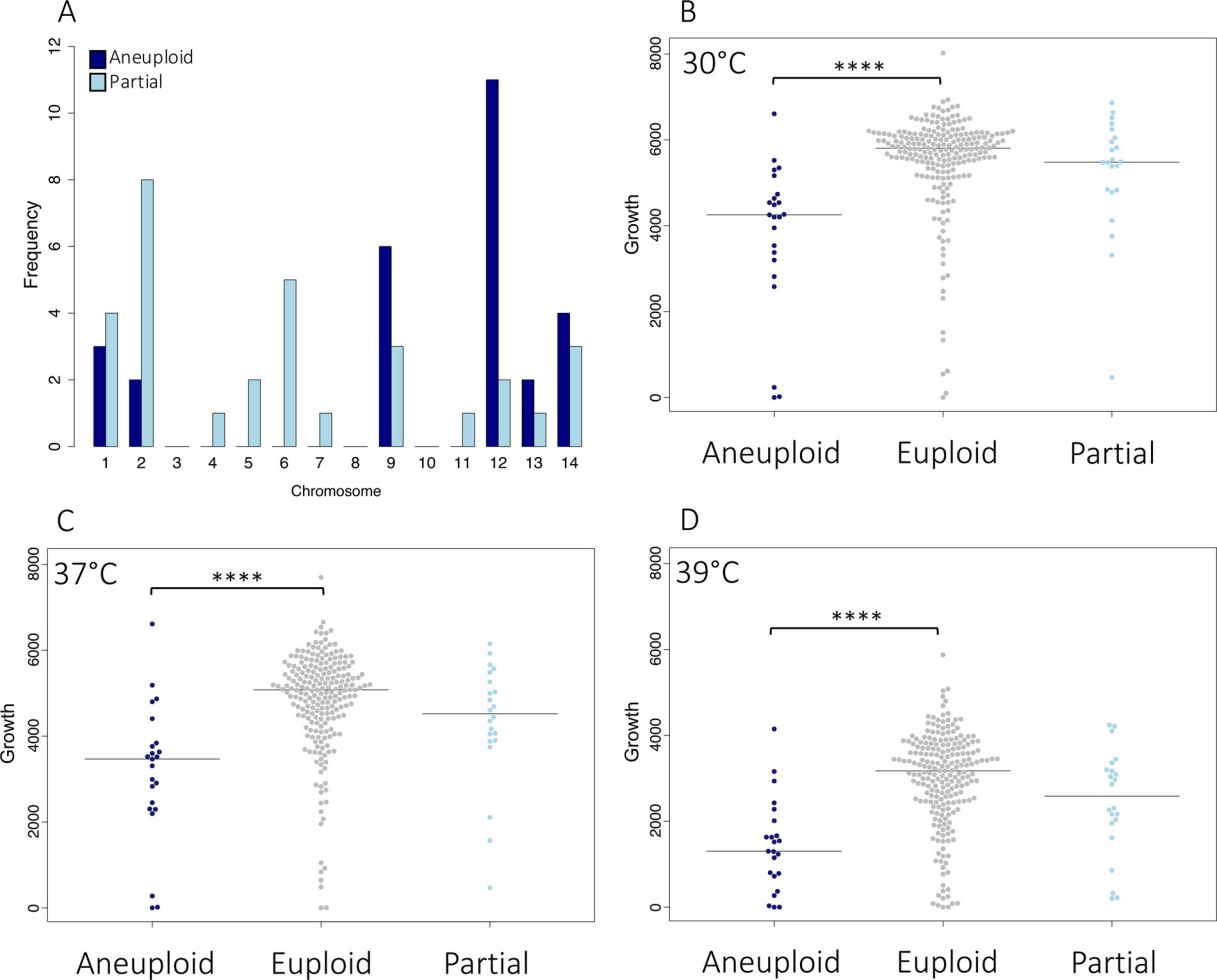
Impact of aneuploidy on growth phenotypes. (A) Whole (aneuploid) and partial chromosomal duplication frequency throughout the population, by chromosome. Colony size (growth) on YPD, by ploidy state at (B) 30°C, (C) 37°C, (D) 39°C.

In addition to SNP and indel mutations, we evaluated the level of chromosome copy number variation across these clinical isolates. We observed both fully and partially duplicated chromosomes, with the most commonly duplicated chromosomes being 12, 9, 14, and 1. Overall, the duplication of an entire chromosome occurs in 8.5% of clinical isolates. Partial duplications, where at least 25% of the chromosome shows continuous duplication, occur most frequently in chromosomes 2 and 6 (15 instances) (Fig. 5A). Aneuploid isolates appear well-distributed throughout this clinical data set (Fig. S5A), suggesting frequent and independent origins for the occurrence of these events *in vivo*.

10.1128/mbio.02626-22.5 FIG S5 Aneuploidy impact on growth for the ACTA and the Desjardins et al. isolates. (A) Whole (aneuploid) and partial chromosomal duplications throughout the population. (B) Colony size (growth) by ploidy state on YPD at 37°C for both the ACTA and the Desjardins et al. isolates. Download FIG S5, PDF file, 0.3 MB. Copyright © 2022 Sephton-Clark et al. 2022 Sephton-Clark et al. https://creativecommons.org/licenses/by/4.0/ This content is distributed under the terms of the Creative Commons Attribution 4.0 International license.

Next, to evaluate the impacts of these large aneuploidies, we compared the growth of aneuploid and euploid isolates. As aneuploidy, specifically that of chromosome 1, has been linked to azole resistance, we first assessed the abilities of these aneuploid isolates to grow in the presence of fluconazole at 64 μg/mL. After 3 days, we observed that 4% (2 isolates, 1 of which showed a chromosome 1 aneuploidy) of the aneuploid isolates were capable of growth under this condition, while 9% (22 isolates) of the euploid isolates were capable of growth on fluconazole. As aneuploidy across a range of chromosomes did not appear to offer a specific advantage to growth on fluconazole, we went on to assess the growth phenotypes of these isolates at a range of temperatures. On rich media (YPD) at 30°C, 37°C, and 39°C, isolates harboring a fully duplicated chromosome showed significantly poorer growth than did euploid isolates (Wilcoxon test, *P* < 5.00E−07) or isolates featuring only a partial chromosomal duplication (Wilcoxon test, *P* < 0.01) (Fig. 5B–D). To determine whether this fitness reduction occurs in both clinical and environmental populations, we carried out a meta-analysis of the data for these isolates with data previously generated using the same assay for a diverse set of clinical and environmental isolates (Fig. S5B) (11). Isolates with aneuploidies present in both data sets displayed a significant reduction in growth on YPD at 37°C (*P* = 2.00E−07), demonstrating that this reduction in fitness holds true for both clinical and environmental isolates across lineages VNI and VNB. We did not observe any significant association between aneuploidy and any single clinical factor; however, we did note that the aneuploid isolates showed a reduced fungal burden in patients (825,928 average CFU/mL), compared to their euploid counterparts (1,318,576 average CFU/mL), though this difference was not statistically significant.

Aneuploidy of specific chromosomes may be advantageous under certain stressors, such as antifungal treatment; however, with optimal nutrients at a range of temperatures, we found that aneuploidy significantly reduces fitness. The modulation of chromosome 1 ploidy has been linked to apoptosis-inducing factor 1 (*AIF1*) (13); however, variants within *AIF1* were not present in this population, suggesting that an alternate mechanism may be responsible for the modulation of the chromosome copy number here.

## DISCUSSION

The phylogenetic analysis of 284 C. neoformans samples from patients with cryptococcosis in Malawi revealed a mixed population dominated by the VNI genetic group, the most commonly observed global lineage of C. neoformans. This population consists of two of the three previously identified VNI lineages (VNIa, and VNIb) (50, 51). VNI isolates are found around the globe and appear relatively clonal, compared to the highly diverse VNB lineage that is often isolated from rural niches, such as mopane trees, in Africa and South America (11, 52–54).

Sugar transporters have previously been identified as under selection in both VNI and VNB isolates from Botswana (11), and we found that they also appear under selection in both VNI and VNB isolates in this population from Malawi. Selection pressures in Cryptococcus likely occur in the environment and provide a coincidental advantage during human infection; for example, transporter gene evolution has been proposed to be driven in part by adaptation to different trees (55). We found inositol, xylose, glucose, lactose, and glucoside transporters under selection. The expansion of inositol transporters in C. neoformans may offer an advantage in both woodland areas and in the CNS, as these environments have abundant inositol (30). Xylose transport is important for C. neoformans capsule production, and the variable xylosylation of an isolate may enable immune evasion (49). The signaling molecule and preferred carbon source, namely, glucose, the precursor of glucoside, is known to regulate hexose transporters that are required for virulence (31) and is a key glycolytic metabolite, a pathway that is required for growth in the CNS (25).

We identified multiple variants that are significantly associated with clinical and growth phenotypes by taking a GWAS approach. However, clinical phenotypes, such as mortality and mental status, did not show a strong association with any variants identified, perhaps due to the complex nature of these clinical characteristics. Additionally, we used a culture-based method to select for isolates from patients. Thus, culture-negative individuals were excluded. As a result, we were unable to detect variants associated with low levels of fungal burden in the CNS. While high fungal burden and mortality are correlated within this data set, additional host factors, such as immune responses, are also known to modulate patient outcomes (56, 57). In this cohort, while CSF burden has a high correlation with infection outcome, there are multiple cases in which the individual either survived or died, despite a high or low initial fungal burden, respectively. These confounding factors may explain why we see a strong association of variants with fungal burden but not with more complex phenotypes, such as mortality. While extensively applied to human data, genome-wide association studies have also been applied to study fungal human pathogens (9–11) and plant-pathogens (58, 59). A major challenge is adapting these association approaches for the population structure of each species; for C. neoformans, while there is recombination within the population, the LD50 values for VNI, VNBI, and VNBII populations are < 50 kb (11). Expanding the sample size for such associations or focusing narrowly on particular genetic groups can help increase the power to detect variants; however, the choice of GWAS approach also needs to be optimized for the population under study through the consideration of the population structure and size.

Through an analysis of the clinical metadata, we found the CSF fungal burden, a measure of the quantity of live yeasts at the site of infection, to be the most strongly associating clinical parameter in carrying out GWAS. We found that a high fungal burden within the CSF of an individual strongly correlated with patient mortality, in accordance with prior work showing that a high fungal load is a predictor of mortality (33–35). Multiple variants were associated with high fungal burden, and when a pair of clinical isolates were tested in a murine model, the isolate containing a frameshift in CNAG_04102 showed increased virulence, compared to its wild-type counterpart. Conversely, when we tested two independent deletions of CNAG_04102 in an H99 background, we did not see this phenotype recapitulated, suggesting that other background specific factors may be at play. Similarly, when we tested an available deletion of another gene impacted by these variants, CNAG_05608, in the KN99 background, we saw reduced virulence in a mouse infection model. However, this was not phenocopied in the H99 background. Furthermore, the variants significantly associated with high fungal burden in CNAG_05608 were exclusively missense variants, which may have altered the protein’s functionality in a way that did not result in such a drastic loss of function as did a deletion strain. Of note, when records were analyzed for the individuals infected with isolates containing missense variants in CNAG_05608, we found that a similar proportion of these patients died, compared to the overall mortality within this cohort (odds ratio of 1.591, *P* value of 0.431).

We found that isolates lacking a functional phosphofructokinase B (CNAG_06033) exhibited slightly accelerated mortality in mice but displayed a reduced CSF load within a rabbit model. We hypothesize that these conflicting findings reveal how Cryptococcus adjusts its energy use during infection in different hosts. Phosphofructokinase plays a key regulatory role in the glycolytic pathway, which appears to be required for optimal virulence in the rabbit model. However, loss-of-function variants in CNAG_06033 were associated with higher fungal burdens in patients and with reduced survival in mice. This later phenotype is consistent with the hypervirulence phenotype that was previously observed when comparing serial isolates from the same patient; a persistent isolate harboring a frameshift in CNAG_06033 showed hypervirulence in a galleria model, compared to the initial infecting isolate (15). Temporal and environmental differences between the human CNS and the rabbit CNS may alter the expression of, and the reliance on, specific metabolic pathways in these two environments. For instance, CNAG_06033 is differentially expressed when comparing Cryptococcus samples taken from human CSF, where it is downregulated, with those from rabbit CSF, where it is upregulated. It is also differentially expressed across the course of infection in the rabbit infection model when comparing days 1 and 4 postinfection in the rabbit CSF. When comparing the cryptococcal transcription of genes in metabolic pathways between rabbit and human CSF samples, those involved in glycogen metabolism appear to be significantly upregulated in human CSF compared to rabbit CSF, and this observation suggests an increased reliance on the metabolism of stored carbon sources for the yeast in a human host (23), hinting that efficient glycolysis may be less necessary for cryptococcal growth in this setting. Within this ACTA cohort, patient isolates containing naturally occurring loss-of-function variants in CNAG_06033 showed similar mortality compared to the overall mortality (odds ratio of 0.774, *P* value of 0.770). These isolates showed no growth defects when grown on YPD at 37°C, and our phosphofructokinase mutant showed a CSF load reduction similar to that observed for the pyruvate kinase (*PYK1*) deletion strain within a rabbit model, likely due to the similar regulatory effects of both enzymes in glycolysis in an acute rabbit model of infection (25). Additionally, loss-of-function variants have previously been identified in CNAG_06033 (PfkB) after *in vivo* human passage (15). Under stress conditions, metabolically heterogeneous yeast populations may emerge (60, 61), and this metabolic diversity might explain the emergence of isolates that are less reliant upon glycolysis for chronic growth within the host, as shutting down a major regulatory gene of glycolysis may allow for more efficient gluconeogenesis, fatty acid use, and beta oxidation for energy (62). In contrast, for growth within the acute rabbit model, Cryptococcus relies upon an intact glycolytic pathway for efficient stress responses and growth in the CNS. These results demonstrate the importance of both the body site and the timing of central metabolic pathways during infection.

This unbiased GWAS approach has allowed us to identify hypothetical proteins implicated in virulence in the absence of additional functional or pathway information. Other systematic studies utilizing RNA-Seq have also identified genes that encode hypothetical proteins that are strong candidates for further study, due to their high expression in CSF (23, 28). While a large proportion of the C. neoformans genes that are annotated as hypothetical proteins are more challenging to study, it is critical that we more widely characterize the roles of all of the genes involved in the pathogenesis of C. neoformans.

We found evidence that ploidy directly impacts the fitness of both clinical and environmental C. neoformans isolates. Aneuploidy has been linked to broad-spectrum stress resistance in *Candida* species (63), and in C. neoformans, disomy of chromosome 1 is known to arise in isolates treated with azoles both *in vitro* and *in vivo* and confers resistance to azoles, such as fluconazole, through an increase in the copy numbers of *AFR1* and *ERG11* (12, 14). Suggested mechanisms for the modulation of chromosome 1 ploidy include regulation via the apoptosis-inducing factor Aif1 (13). However, we did not find evidence for Aif1 disruption in these isolates. Specific impacts of disomy have also been noted for chromosome 13, the disomy of which results in reduced melanization (20). In S. cerevisiae, disomy of select chromosomes also results in reduced proliferation rates (64). While the reduction in fitness that we observed does not seem specific to any particular chromosome, the questions of how and why ploidy appears subject to change under stressful conditions and whether the most frequently observed aneuploidies confer a specific advantage are intriguing and require further study.

By combining genetic, *in vitro*, clinical data and animal model validation, we gain insights into the impact of naturally occurring genetic variation and its implication for infection outcomes. As whole-genome sequencing on an ever-larger scale becomes more accessible, and as association techniques for fungal populations grow in sophistication, so, too, will our power to detect functionally relevant genetic variation across cryptococcal populations. Combining data from large pan-African clinical trials with approaches that leverage both fungal and human variant identification, we can further dissect the interactions between pathogen and host genetics. Together, this will enable a better understanding of how these variations impact the ability of Cryptococcus to adapt to and thrive in the wide range of environments in which they find themselves.

## MATERIALS AND METHODS

### Sample preparation and sequencing.

Clinical cryptococcal isolates derived from patient CSF subculture were procured through the Antifungal Combinations for Treatment of Cryptococcal Meningitis in Africa Trial (ACTA) (24). Repeat cultures and duplicates were excluded. Collected strains were grown overnight in 10 mL of YPD at 30°C and 225 rpm. Then, genomic DNA was extracted for sequencing using a MasterPure Yeast DNA Purification Kit, as described by Desjardins et al. (11). The DNA was sheared to 250 bp using a Covaris LE instrument and adapted for Illumina sequencing as described by Fisher et al. (65). Libraries were sequenced on a HiSeq X10, generating 150 bp paired reads (minimum average coverage of 100×).

### Data processing and variant calling.

To determine sample species, reads were first aligned to a composite pan-Cryptococcus genome, consisting of reference genomes for Cryptococcus neoformans var. *grubii* H99, Cryptococcus neoformans var. *neoformans* JEC21, and representative genomes for lineages VGI, VGII, VGIIIa, VGIIIb, VGIV, and VGV of Cryptococcus gattii (66–69). To identify variants for C. neoformans species, reads were aligned to the Cryptococcus neoformans var. *grubii* H99 reference genome (GCA_000149245.3) (67) with BWA-MEM version 0.7.17 (70). GATK v4 variant calling was carried out as documented in our publicly available cloud-based pipeline (71) (https://github.com/broadinstitute/fungal-wdl/tree/master/gatk4). After calling, variants were filtered on the following parameters: QD < 2.0, QUAL < 30.0, SOR > 3.0, FS > 60.0 (indels > 200), MQ < 40.0, GQ < 50, alternate allele percentage = 0.8, and DP < 10. Variants were annotated with SNPeff, version 4.3t (72).

### Population genomic analysis.

A maximum likelihood phylogeny was estimated using 72,258 segregating SNP sites that were present in one or more isolates, allowing ambiguity in a maximum of 10% of samples, with RAxML version 8.2.12 GTRCAT rapid bootstrapping (73). The maximum likelihood phylogeny was visualized with ggtree (R 3.6.0) and was rooted to the VNII isolates. The isolate lineage was identified based on phylogenetic comparisons with previously typed isolates that were reported by Desjardins et al. (11). To estimate linkage disequilibrium (LD) decay, vcftools version 0.1.16 was used to calculate the LD values for 1,000 bp windows, with a minimum minor allele frequency of 0.1 and the –hap-r2 option. Region deletions and duplications were identified using CNVnator v0.3 (significant instance e value <0.01) (74). To identify regions under selection, a composite likelihood ratio analysis was performed using PopGenome (R 3.5.0, PopGenome 2.7.5) per chromosome with 5 kb windows (75). The top 5% scoring regions (centromeric regions excluded) were tested for enrichment via a hypergeometric test with the false discovery rate correction. Large duplications and aneuploidies were visualized using funpipe (coverage analysis) version 0.1.0 (https://github.com/broadinstitute/funpipe).

### Genome-wide association studies.

An association analysis between clinical data, *in vitro* phenotypes, and variants was carried out using PLINK v1.08p formatted files and GEMMA version 0.94.1 (76) (options: centered relatedness matrix gk 1, linear mixed model), as previously described (11). Variants were considered in two scenarios: one in which rare variants (present in <5% of the population) were collapsed by gene, and another in which loss-of-function variants (SNPeff impact high) were considered independently. Significant variants were those with a test score of <1.00E−6.

### Clinical data analysis.

Deidentified clinical metadata detailing the CSF fungal burden (CFU/mL), fungal clearance (EFA), patient mortality, and Glasgow coma score were provided by investigators, with these parameters being determined as previously described (24). The correlations between the clinical parameters were determined in R 3.6.0 with the Pearson correlation coefficient, Spearman’s rank correlation coefficient, point-biserial correlation, or phi coefficient. Survival curves were generated using Prism v9.1.0, and statistics were computed in R 3.6.0.

### *In vitro* phenotyping.

Strains were grown at 30°C for 2 to 5 days. For each strain, a single colony was selected and added to 96-well microtiter plates that contained 200 μL of YPD broth. Each 96-well plate contained 6 control strains (H99 and deletion mutants of *LAC1*, *MPK1*, *CNA1*, *RAS1*, and *HOG1*) and a YPD control. The 96-well plates were incubated for 1 to 2 days at 30°C. The colonies were pin replicated into 384-well microtiter plates that contained 80 μL of YPD broth in each well. The 384-well plates were incubated for 1 to 2 days at 30°C. They were then pinned onto one-well solid agar plates in duplicate using a BM5-BC Colony Processing Robot (S & P Robotics) in 1536 array format. In three separate biological replicates, the isolates were grown at 30°C, 37°C, and 39°C on YPD agar. Isolates were also pinned onto YPD + 10 μg/mL fluconazole and YPD + 64 μg/mL fluconazole. Images were captured after approximately 24, 48, and 72 h. The colony size at 48 h was determined using gitter (77) and was used to assess growth.

### Gene deletion strains.

The strains used for the animal studies and the primer sequences used are listed in Table S1. KN99alpha (CM026) was used as the reference wild-type strain for deletions obtained from a genome-wide Cryptococcus deletion library (45). Multiple independent deletion strains were generated in wild-type C. neoformans strain H99 (*cnag_05608Δ*, *cnag_04102Δ*, *cnag_06033Δ*) for this study. 3 DNA fragments were amplified via polymerase chain reaction (PCR): approximately 0.7 to 1 kb of the 5′ flank sequence, the nourseothricin (NAT) drug selection cassette amplified from pAI3 (78), and 0.7 to 1 kb of the 3′ flank sequence were prepared for each gene. The PCR products were gel extracted using a NucleoSpin Gel and PCR Clean-up Kit (Macherey-Nagel). Next, the PCR products were cloned into pUC19 using a NEBuilder HiFi DNA Assembly and transformed into Escherichia coli. Positive plasmids were confirmed via PCR. For biolistic transformation, 2 μg of plasmid was transformed into the C. neoformans strain H99 as previously described (79), with a slight modification that the yeast cells were recovered on YPD containing 0.75 M sorbitol and 0.75 M mannitol. The cells were allowed to recover for 2.5 h before they were transferred to the selective medium, YPD + 100 μg/mL NAT. Positive transformants were confirmed via PCR.

10.1128/mbio.02626-22.7 TABLE S1 Wild-type and mutant strains used in this study. Download Table S1, XLSX file, 0.1 MB. Copyright © 2022 Sephton-Clark et al. 2022 Sephton-Clark et al. https://creativecommons.org/licenses/by/4.0/ This content is distributed under the terms of the Creative Commons Attribution 4.0 International license.

### Capsule production.

To evaluate the capsule size, capsule inducing medium (10% Sabouraud broth in 50 mM MOPS [pH 7.3]) was used as previously described (80). 5 mL of capsule-inducing medium were inoculated with a single freshly streaked yeast colony and were grown in an incubator shaker (225 rpm) for approximately 24 and 48 h. India ink was used as a counterstain at a 1:5 ratio (ink:cell suspension). Images were captured via microscopy (Zeis Axio Imager 1). The cell body and capsule sizes were measured in ImageJ V1.53a.

### Murine model of infection.

C. neoformans strains were grown in YPD broth at 30°C in a shaking incubator (220 rpm) for 24 h, centrifuged, and washed twice in phosphate-buffered saline (PBS). Yeast cell counts were determined using a T4 cell counter (Nexcelom). For gene deletion strains, five male, 22 to 24 g CD-1 mice (Charles River Laboratories) were infected with approximately 5 × 10^4^ yeast cells per mouse via oropharyngeal aspiration while under isoflurane anesthesia. For the clinical isolates, 15 male, 22 to 24 g CD-1 mice (Charles River Laboratories) were infected with approximately 5 × 10^4^ yeast cells per mouse via oropharyngeal aspiration while under isoflurane anesthesia. The mice were monitored and weighed daily. Mice with a total body weight loss of ≥20% or exhibiting behavioral, neurological, or respiratory symptoms were sacrificed, following the IACUC guidelines, via CO_2_ asphyxiation. The Kaplan-Meier survival plots and analyses (the log-rank test) were completed using Prism software v9.1.0., GraphPad Software. A *P* value of ≤0.05 was considered to be indicative of a statistically significant result. Outliers were excluded based on a ROUT analysis that was performed using Prism software v9.1.0., GraphPad Software.

### Rabbit model of infection.

To assess the fitness and virulence of the deletion strains in rabbit CSF, 3 New Zealand White male rabbits (2.3 to 4.7 kg) were inoculated intracisternally with 300 μL of approximately 1 × 10^8^ cells. The animals were sedated with ketamine and xylazine for inoculation and serial CSF cisternal taps. The rabbits were treated daily with hydrocortisone acetate (2.5 mg/kg) via intramuscular injections, starting 1 day prior to the inoculation of the yeast. Cisternal taps were performed on days 3, 7, and 10, and this was followed by the serial dilution of the CSF and the enumeration of colonies. The time series fungal burden data were then assessed with the estimated marginal means of linear trends via repeated measures analysis in R v3.6.1.

### Animal studies.

The animal experiments were performed in compliance with the Animal Welfare Act, the *Guide for the Care and Use of Laboratory Animal*s (81), and the Duke Institutional Animal Care and Use Committee (IACUC).

### Ethics.

The ACTA trial from which the isolates described here were collected had ethical approval from the London School of Hygiene and Tropical Medicine Research Ethics Committee and by all of the site national research ethics committees and regulatory bodies. Deidentified clinical metadata (fungal burdens, fungal clearances, patient outcomes) were provided by the investigators for the analyses performed here.

### Data availability.

The isolate sequence data can be accessed in NCBI via accession number PRJNA764746.
