# Supplementary material for: Genomic Variation across a Clinical Cryptococcus Population Linked to Disease Outcome
Source: mBio. 2022 Nov 10;13(6):e02626-22. doi: 10.1128/mbio.02626-22 (PMC9765290; doi:10.1128/mbio.02626-22)
Supplement: FIG S1 [file mbio.02626-22-s0005.pdf]

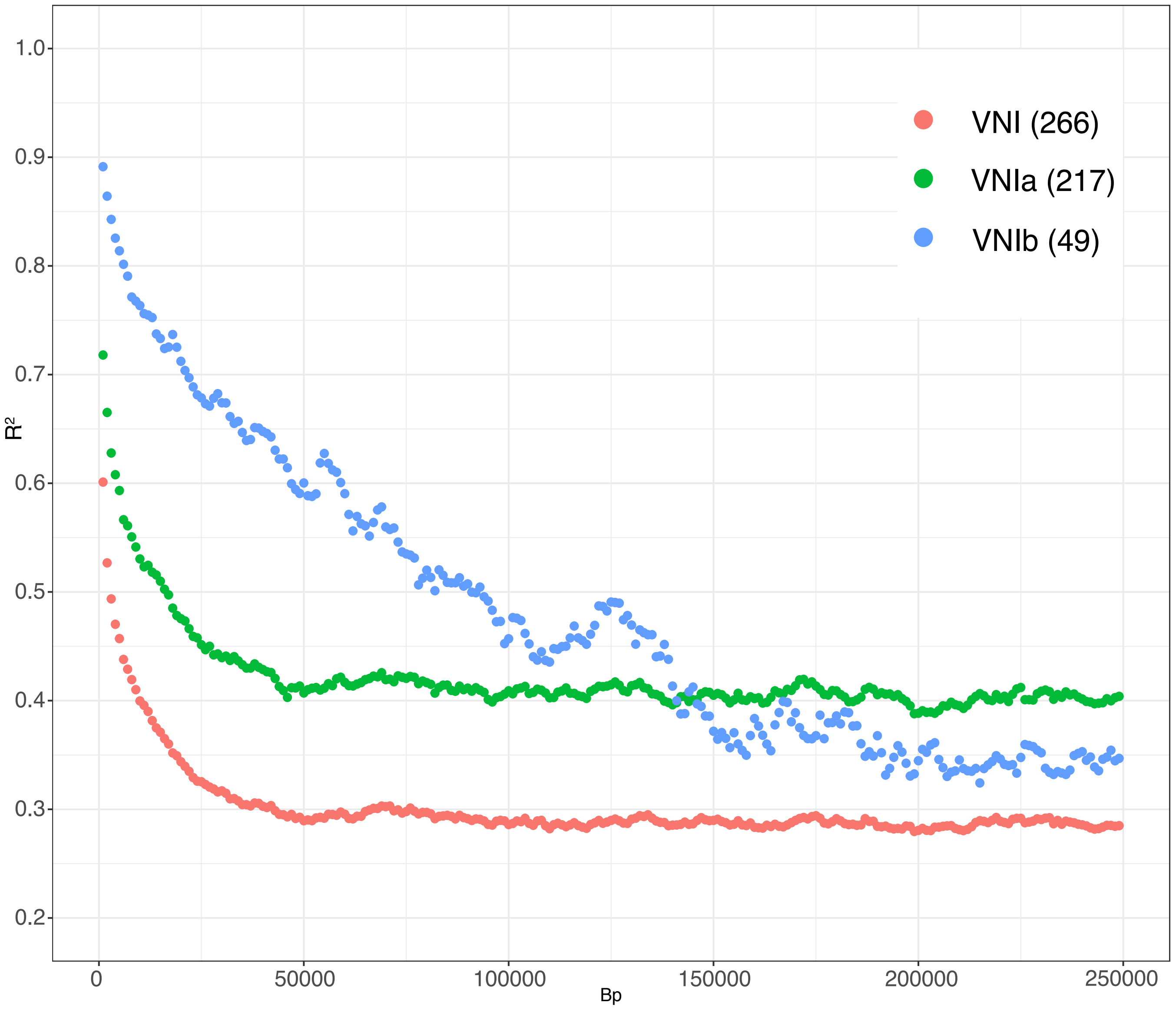

Supplemental Figure 1. Linkage disequilibrium decay over 250kb for lineages VNIa, VNIb, and VNI (VNIa + VNIb). VNI shows 50% LD decay in 30kb.
