# Supplementary material for: Genomic Variation across a Clinical Cryptococcus Population Linked to Disease Outcome
Source: mBio. 2022 Nov 10;13(6):e02626-22. doi: 10.1128/mbio.02626-22 (PMC9765290; doi:10.1128/mbio.02626-22)
Supplement: FIG S2 [file mbio.02626-22-s0006.pdf]

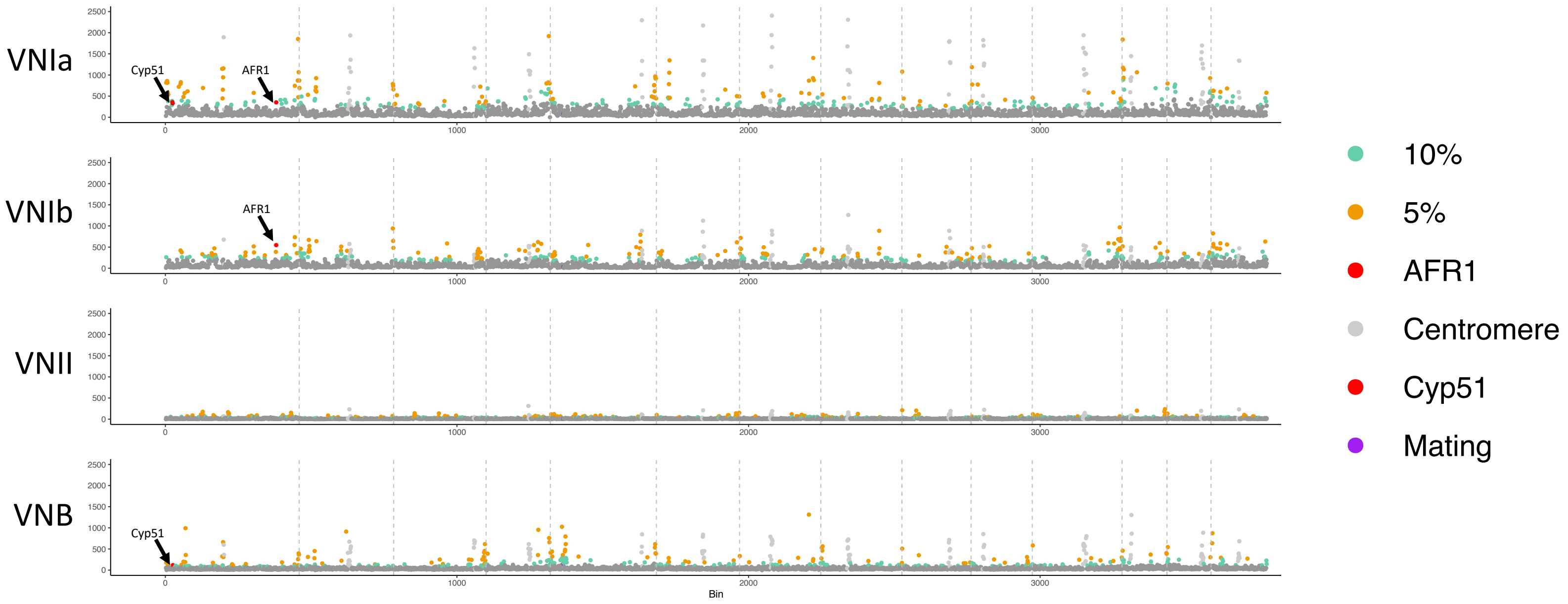

Lineage	Enriched Category (GO)	Gene Functions	Corrected P Value
VNIa	Carbohydrate Transport	Inositol Transport, Glucose and Glucoside Transport	1.0765E-3
VNII	Nucleotide Excision Repair	*UVE1, RAD23, RHP42, ERCC2*	6.7712E-3
VNB	Carbohydrate Transport	Inositol Transport, Glucoside Transport, Galactose Transport	8.6025E-4

Supplemental Figure 2. Regions under selection per lineage, determined by CLR analysis. Highlighted regions are in the top 5% (orange) or top 10% (green) of all regions. Centromeric regions are highlighted in light grey. Region enrichment via hypergeometric testing for GO categories by lineage, with contributing genes and adjusted p-values given.
