# Supplementary material for: Genomic Variation across a Clinical Cryptococcus Population Linked to Disease Outcome
Source: mBio. 2022 Nov 10;13(6):e02626-22. doi: 10.1128/mbio.02626-22 (PMC9765290; doi:10.1128/mbio.02626-22)
Supplement: FIG S3 [file mbio.02626-22-s0007.pdf]

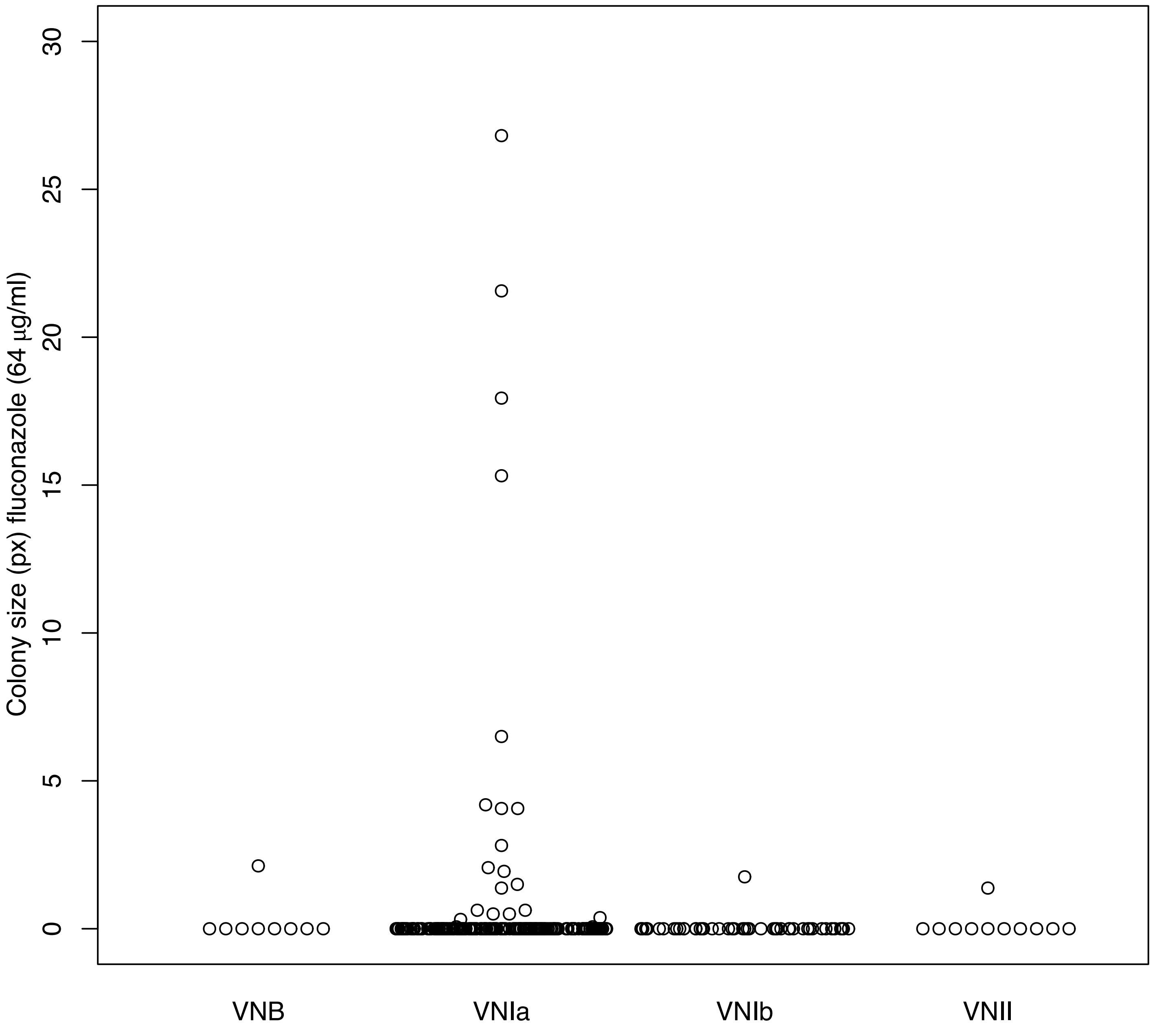

Supplemental Figure 3. Growth on fluconazole by lineage. Colony size (area, px) of isolates grown on fluconazole 64ug/ml for 3 days.
