# Supplementary material for: Genomic Variation across a Clinical Cryptococcus Population Linked to Disease Outcome
Source: mBio. 2022 Nov 10;13(6):e02626-22. doi: 10.1128/mbio.02626-22 (PMC9765290; doi:10.1128/mbio.02626-22)
Supplement: FIG S6 [file mbio.02626-22-s0010.pdf]

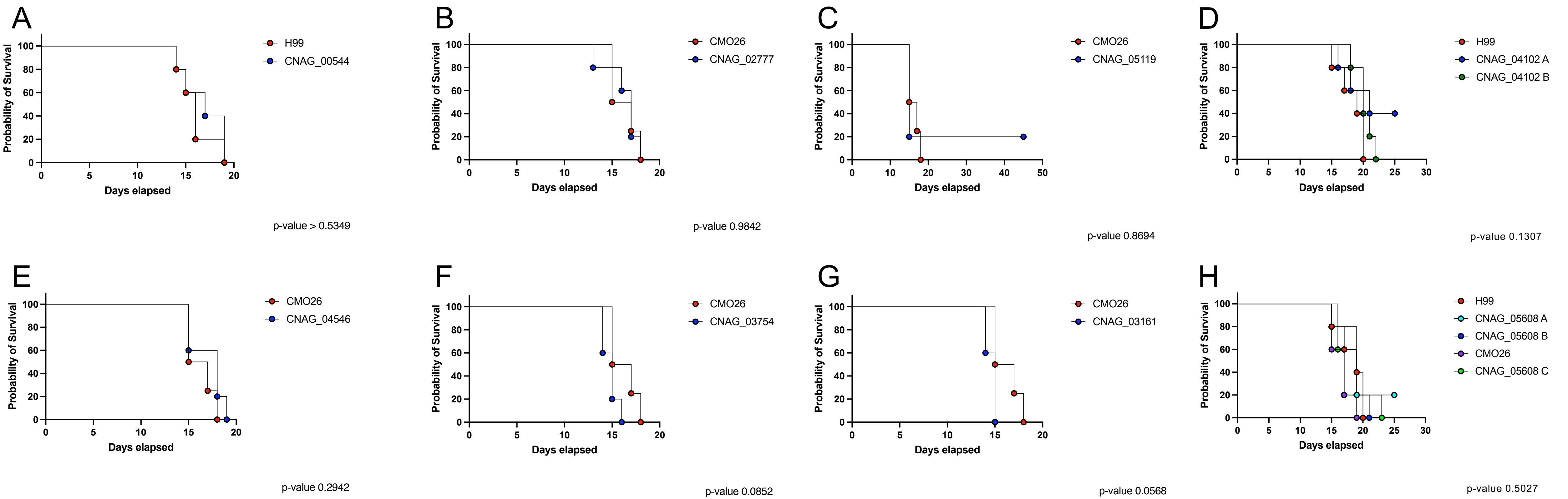

Supplemental Figure 6. Survival of mice infected with parental strain (H99/CMO26) or deletion strain. Five CD-1 mice were infected with approximately $5x10^4$ CFU by oropharyngeal aspiration per strain. A) Survival of mice infected with parental strain (H99) and a CNAG_00544 mutant strain. B) Survival of mice infected with parental strain (CMO26) and a CNAG_02777 mutant strain. C) Survival of mice infected with parental strain (CMO26) and a CNAG_05119 mutant strain. D) Survival of mice infected with parental strain (H99) and two independent CNAG_04102 mutant strains. E) Survival of mice infected with parental strain (CMO26) and a CNAG_04548 mutant strain. F) Survival of mice infected with parental strain (CMO26) and a CNAG_03754 mutant strain. G) Survival of mice infected with parental strain (CMO26) and a CNAG_03161 mutant strain. H) Survival of mice infected with parental strain (H99/CMO26) and three independent CNAG_05608 mutant strains in the CMO26 background (C) and the H99 background (A, B).
