# Supplementary material for: Genomic Variation across a Clinical Cryptococcus Population Linked to Disease Outcome
Source: mBio. 2022 Nov 10;13(6):e02626-22. doi: 10.1128/mbio.02626-22 (PMC9765290; doi:10.1128/mbio.02626-22)
Supplement: FIG S4 [file mbio.02626-22-s0008.pdf]

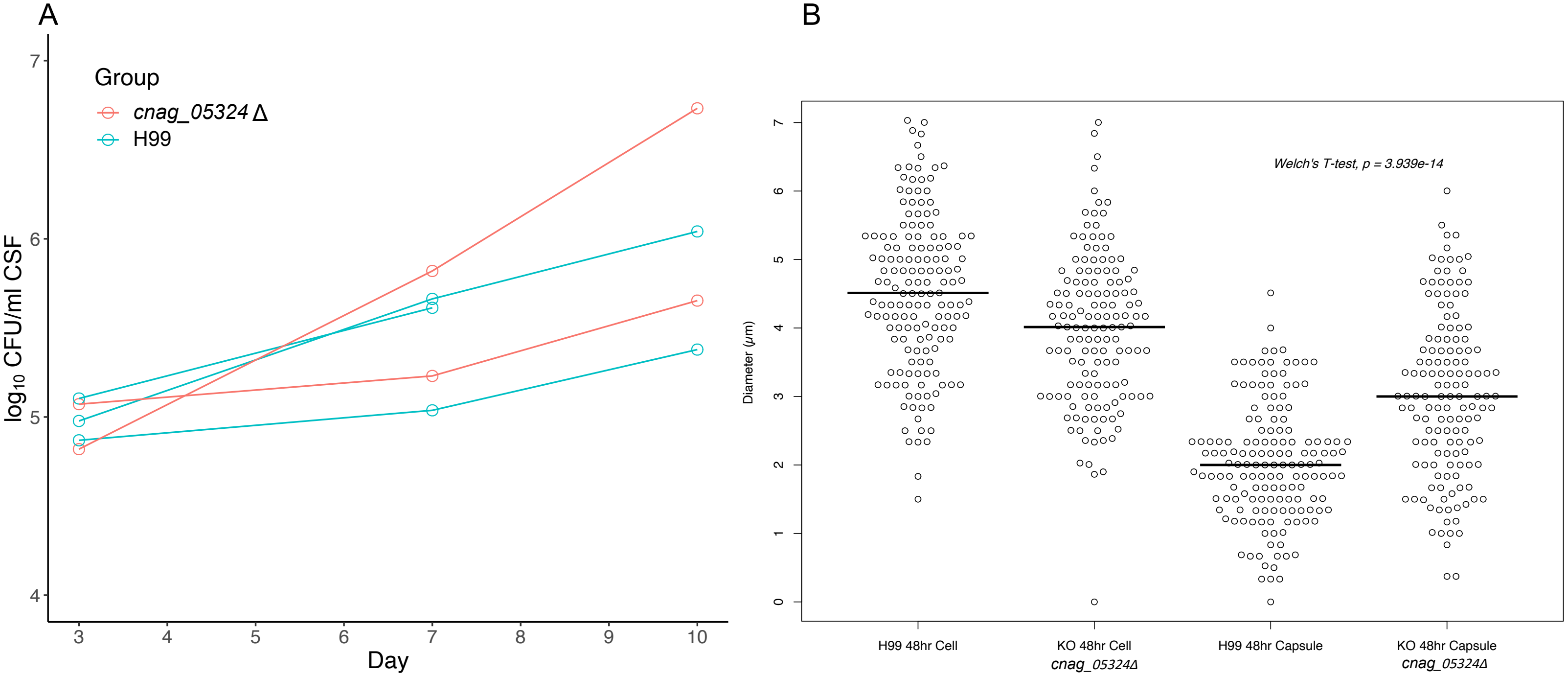

Supplemental Figure 4. Capsule and virulence regulation of CNAG_05324. A) Rabbit CSF load, Log10(CFU/ml), on days 3, 7, and 10, per rabbit (individual lines) infected with either H99 (blue) or the CNAG_05324 deletion strain (pink). B) Cell size and capsule diameter at 48 hours growth in capsule inducing media for H99 and the CNAG_05324 deletion strain.
