# Supplementary material for: Genomic Variation across a Clinical Cryptococcus Population Linked to Disease Outcome
Source: mBio. 2022 Nov 10;13(6):e02626-22. doi: 10.1128/mbio.02626-22 (PMC9765290; doi:10.1128/mbio.02626-22)
Supplement: FIG S5 [file mbio.02626-22-s0009.pdf]

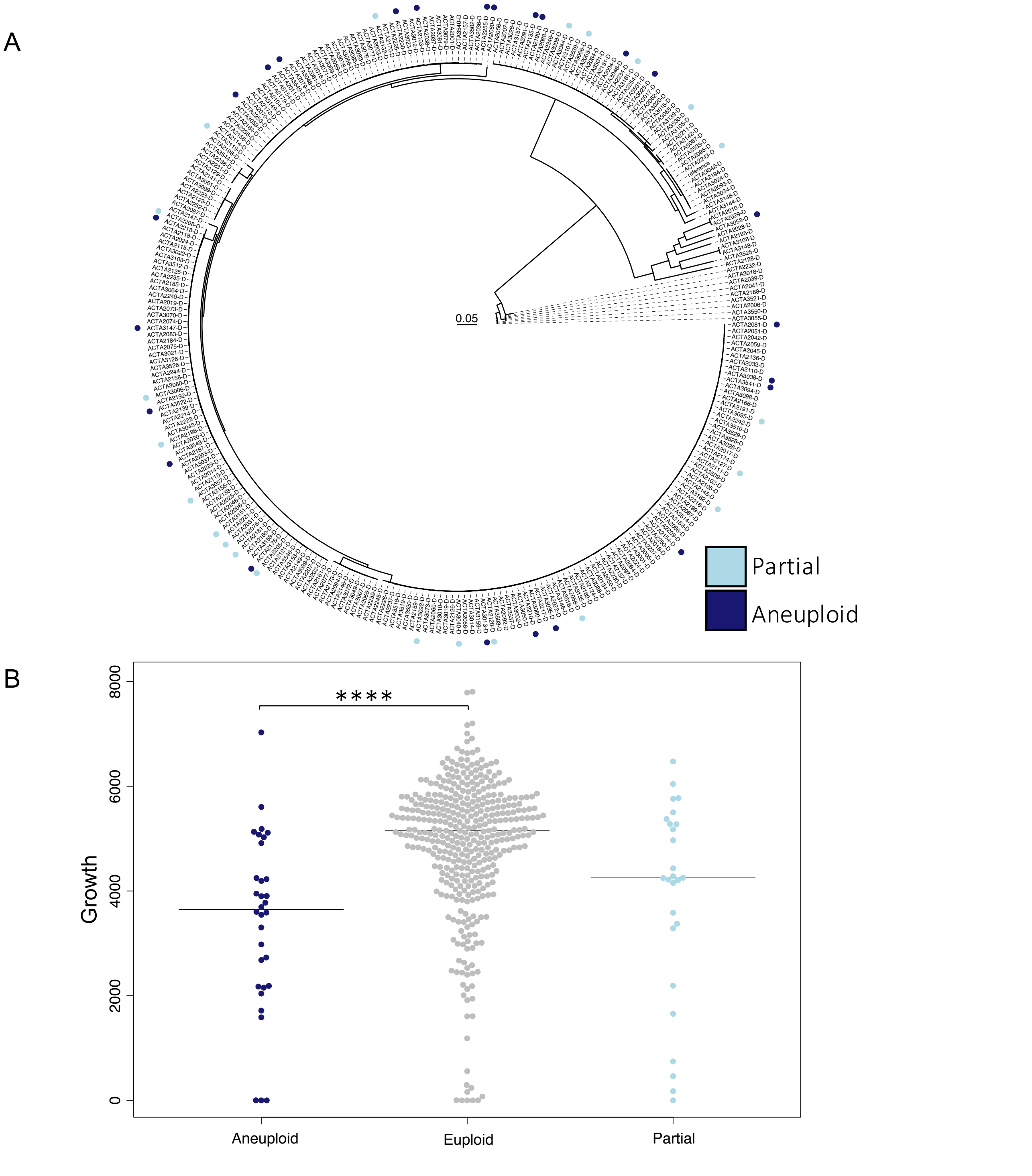

Supplemental Figure 5. Aneuploidy impact on growth for ACTA and Desjardins et al isolates. A) Whole (aneuploid) and partial chromosomal duplications throughout the population. B) Colony size (growth) by ploidy state on YPD at 37°C for both ACTA and Desjardins et al isolates.
